# Optimization of loading protocols for tissue engineering experiments

**DOI:** 10.1038/s41598-022-08849-y

**Published:** 2022-03-24

**Authors:** Yann D. Ladner, Angela R. Armiento, Eva J. Kubosch, Jess G. Snedeker, Martin J. Stoddart

**Affiliations:** 1 AO Research Institute Davos, Clavadelerstrasse 8, 7270 Davos Platz, Switzerland; 2 Institute for Biomechanics, ETH Zurich, Lengghalde 5, 8008 Zurich, Switzerland; 3 Department of Orthopedics and Trauma Surgery, Medical Center-Albert-Ludwigs-University of Freiburg, Faculty of Medicine, Albert-Ludwigs-University of Freiburg, 79106 Freiburg, Germany; 4 Department of Orthopaedics, University Hospital Balgrist, Lengghalde 5, 8008 Zurich, Switzerland

**Keywords:** Mesenchymal stem cells, Tissue engineering, Stem-cell biotechnology

## Abstract

Tissue engineering (TE) combines cells and biomaterials to treat orthopedic pathologies. Maturation of de novo tissue is highly dependent on local mechanical environments. Mechanical stimulation influences stem cell differentiation, however, the role of various mechanical loads remains unclear. While bioreactors simplify the complexity of the human body, the potential combination of mechanical loads that can be applied make it difficult to assess how different factors interact. Human bone marrow-derived mesenchymal stromal cells were seeded within a fibrin-polyurethane scaffold and exposed to joint-mimicking motion. We applied a full factorial design of experiment to investigate the effect that the interaction between different mechanical loading parameters has on biological markers. Additionally, we employed planned contrasts to analyze differences between loading protocols and a linear mixed model with donor as random effect. Our approach enables screening of multiple mechanical loading combinations and identification of significant interactions that could not have been studied using classical mechanobiology studies. This is useful to screen the effect of various loading protocols and could also be used for TE experiments with small sample sizes and further combinatorial medication studies.

## Introduction

Tissue engineering (TE) aims to find optimal solutions to improve patient outcomes by combining specific cellular and material-based approaches selected from a vast variety of options. TE applied to the musculoskeletal field has placed much hope on mesenchymal stromal/stem cells (MSCs) due to their multilineage potential and secretory profile. Under specific stimuli, MSCs can differentiate into chondrocytes and osteoblasts, therefore representing a valuable tool for cell-based therapies targeting orthopedic pathologies, such as cartilage and bone injuries. Articular cartilage defects present a higher risk of post-traumatic osteoarthritis and thereby increase health-care cost and burden. Similarly, bone defects can result in delayed or non-union in up 20% of the cases despite continuous advancement in the field of bone repair^1^. Biomaterials provide support for cell adhesion, proliferation and differentiation and could thereby enhance the body's reparative response when implanted. Notably, the mechanical stimulation experienced by the MSCs is a key determinant of their differentiation^2–4^. Therefore, not only should the biomaterials provide mechanical support but also transmit the natural mechanical forces within the body to the cells. While a plethora of different MSCs-based strategies in combination with a multitude of biomaterials and have been developed, their clinical translation is still very limited. Despite an enormous body of scientific literature featuring a wide array of cellular, biomaterial, and mechanical stimulation strategies, it is still unclear how mechanical stimulation applied to MSCs affects their differentiation.

One example of a cartilage treatment method is microfracture, a bone marrow-stimulating technique that was developed by Steadman in the 1980s^5^. During this arthroscopic technique, the loose cartilage within the injury is removed and small perforations into the subchondral bone are created to allow for the migration of bone marrow elements, including MSCs, into the defect. In this newly formed environment, the MSCs can be directed towards chondrogenesis and replace the lost cartilage. Yet, the new tissue predominantly resembles fibrocartilage instead of hyaline cartilage, leading to a loss in functionality^6,7^. Steadman accentuates the importance of an appropriate rehabilitation protocol after surgery and believes that an ideal physical environment is necessary to differentiate the MSCs within the recruited bone marrow towards chondrogenesis^8^. However, it is still unclear what exact mechanical stimulation the MSCs need to experience to form hyaline cartilage.

Prior studies have shed light on how exogeneous administration of growth factors can induce chondrogenesis in MSCs in vitro^9,10^. Work from our group has shown that mechanical stimulation alone is sufficient to induce in vitro chondrogenesis, potentially obviating the need for exogenous growth factors^11^. Use of a joint-mimicking multiaxial loading bioreactor results in the activation of latent transforming growth factor β1 (TGF-β1) secreted by MSCs seeded within a porous scaffold. TGF-β is a growth factor that is cardinal in driving chondrogenesis^9^. Experiments using a hyaluronan-based hydrogel have suggested that TGF-β activation might be material dependent, with lower amounts being detected in the softer hydrogel compared to a stiffer porous scaffold^12^. Another chondrogenic inducer in the TGF-β superfamily is bone morphogenetic protein 2 (BMP2). It has been previously shown that tensile strain can upregulate BMP2 gene expression, however, its mechanical regulation remains unclear^13^. It is of note that BMP2 is also a potent osteogenic inducer and is therefore also interesting for bone healing^14,15^. Mechanical stimulation is also associated with an increase in nitric oxide (NO), a possible marker for cellular stress^16–18^.

Bioreactor-based experiments offer a unique opportunity to study applied load under standardized conditions. However, the experiments are both material and time consuming, and testing a multitude of different biomaterials and loading factors (parameters), such as magnitudes and frequencies, proves difficult within a single experiment^2^. Experimentation strategies such as Best Guess and One Factor at a Time (OFAT) are inadequate in finding optimal factor combinations that utilize the optimal levels (settings). Best Guess approaches are heavily reliant on prior knowledge, can continue for a long time and tempt the experimenter to stop testing once they obtain an acceptable result that not necessarily represents the best solution. OFAT methods start from baseline levels for each factor and alter only one factor while keeping the remaining factors constant. This approach cannot capture possible interactions between factors, meaning the experimenter would not know whether one factor leads to a different response depending on different levels of another factor^19^. We therefore hypothesized that a factorial approach used in a branch of applied statistics called design of experiments (DOE) could be utilized. A full factorial experiment allows several factors to be simultaneously compared at multiple levels, to evaluate main and interaction effects. The main effects describe the individual effects of each factor independent of the other factors, whereas the interaction effects depend on the effect of the combination of multiple factors. Furthermore, factorial experiments make full use of their design in a way that each experimental run contributes to the calculation of the main and interaction effects. Combined with contrast analysis, a statistical method where sets of means (in contrasts) are compared to one another according to preplanned hypotheses, this is a powerful tool for studying multiple groups in parallel. Contrasts act like spotlights that illuminate distinct features of the data as opposed to analysis of variances (ANOVA) that acts like a background lighting, which do not provide enough light to observe the details^20^.

As a first step, we focused on the direct role of mechanical stimulation. The present work aims to elaborate whether a methodological approach combining a full factorial DOE and contrasts can be used to screen different mechanical parameters and their complex interactions on MSC differentiation within a biomaterial in an efficient and concise manner. Various combinations of shear and compression were applied. In addition, two different loading counterfaces, a ball or a cylinder, were compared.

This whole methodological framework could be used in regenerative medicine and tissue engineering to efficiently investigate the relative role of multiple factors such as age, inflammation, or optimal mechanical loading in biomaterials, by testing as many different conditions as possible, while keeping replication to a minimum. And by reducing replication, the sample size remains lower, and therefore the number of cells needed in the high cell density constructs needed for cartilage tissue engineering.

## Materials and methods

### Cell isolation, cell culture and scaffold seeding

Human MSCs were isolated with and according to full ethical approval (Ethik-Kommission der Albert-Ludwigs-Universität Freiburg, EK-326/08) and written informed donor consent from bone marrow aspirates of three donors (two males aged 30 and 33; one female aged 57) using Ficoll (Histopaque-1077, Sigma-Aldrich) density gradient and plastic adhesion^21^. All experiments were performed in accordance with relevant regulations. The MSCs were expanded in Minimum Essential Medium alpha (αMEM, Gibco) supplemented with 10% (v/v) fetal bovine serum (Sera Plus, Pan Biotech), 1% (v/v) Penicillin–Streptomycin (P/S, 100 U/mL penicillin and 100 μg/mL streptomycin, Gibco) and 5 ng/mL recombinant human fibroblast growth factor basic protein (FGF-b, Fitzgerald Industries International) until passage 4. Thereafter, 4.5 × 10^6^ cells were seeded into a cylindrical fibrin-poly(ester-urethane) (fibrin-PU) scaffold (average pore size between 150 and 300 μm) with a thickness of 4 mm and a diameter of 8 mm according to previous protocols^22,23^. Briefly, the cells were resuspended in 33.3 mg/mL fibrinogen (Baxter, Austria) and added to the sterile lid of a 1.5-mL Eppendorf tube. An equal volume of a 1 U/mL thrombin solution was then mixed with the fibrinogen-cell solution and the porous scaffold was pressed into the lid. Repeated compression of the scaffold using tweezers allowed for the influx of the cell suspension into the pores. Afterwards, the scaffolds were transferred to an incubator (37 °C) for 40 min. Another 0.5 × 10^6^ cells were suspended in Dulbecco’s modified Eagle medium 4.5 g/L glucose (DMEM, Gibco) and seeded on top of the scaffolds with subsequent incubation for 1 h, as originally described by Gardner et al.^23^. The scaffold samples were cultured in chondropermissive medium (CpM) consisting of Dulbecco’s modified Eagle medium 4.5 g/L glucose (DMEM, Gibco), 0.11 g/L sodium pyruvate (Sigma-Aldrich), 50 μg/mL l-ascorbic acid 2-phosphate sesquimagnesium salt hydrate (Sigma-Aldrich), 100 nM dexamethasone (Sigma-Aldrich), Corning ITS + Premix (6.25 µg/mL human recombinant insulin, 6.25 µg/mL human natural transferrin, 6.25 ng/mL selenious acid, 1.25 mg/mL bovine serum albumin, 5.35 µg/mL linoleic acid, ThermoFisher), 1% non-essential amino acids (Gibco), 1% (v/v) P/S and 5 μM 6-aminocaproic acid (Sigma-Aldrich). The culture conditions were kept constant at 37 °C, 5% CO_2_ and 95% rH.

### Mechanical loading

**Figure 1:**
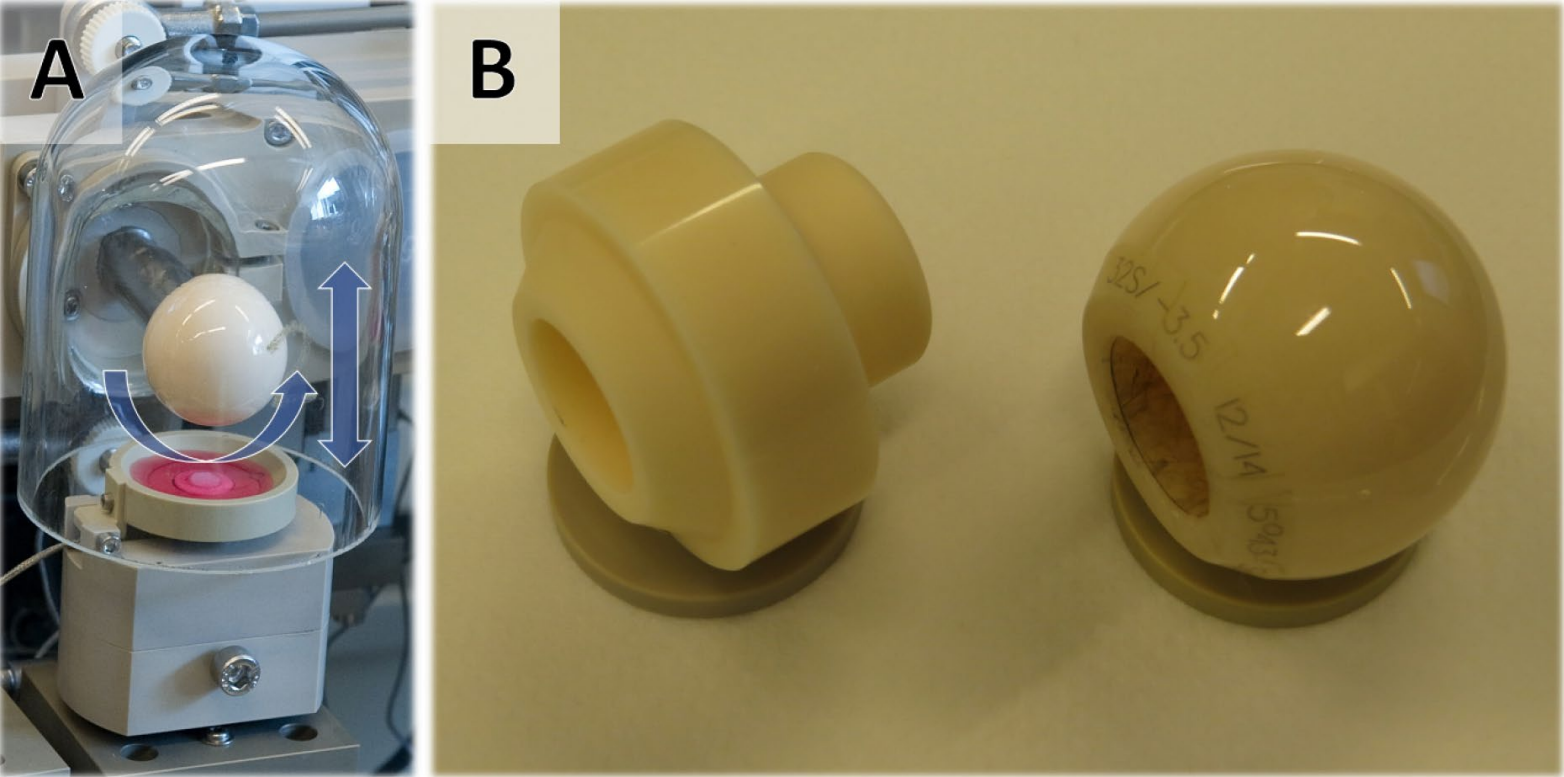
Multiaxial bioreactor and counterfaces. (**A**) Image of a loading station within the custom bioreactor. Arrows indicate rotational and vertical movement of the counterface. (**B**) Image of the two counterface types. Left: cylinder; right: ball.

Scaffolds were loaded according to different loading protocols in a previously described multi-axial load bioreactor system (Fig. 1A)^24^. All the samples were loaded 1 h per day for 10 days over a culture period of 12 days. Different combinations of three factors, namely type of counterface, shear frequency and compressive strain were used to investigate how the factors' interaction influence biomarker secretion. Two types of counterfaces were used to apply the mechanical load: a cylinder and a ball (Fig. 1B). While the ball had already been used in previous studies^11,22,23,25,26^, by contrast, a cylindrical counterface was developed under the assumption that the larger surface of the cylinder would increase surface shear and thereby amplify latent TGF-β1 activation. Two different settings (levels) were used each for the shear frequency (0.2 and 1 Hz) and for the compressive strain (5% and 20%) (Table 1). The shear factor comprised a ± 25° oscillatory rotation of the counterface at different frequencies. Compression comprised a cyclic vertical translational movement of the counterface at different strains. The frequency of compression was fixed at 1 Hz and the fixed static pre-strain amounted to 0.4 mm. To account for the influence of counterface type, center points were included for both cylinder and ball counterface. The setting at 0.6 Hz shear frequency and 10% compressive strain represented the center point that demonstrates whether the relationship between factors is linear and to ensure process stability. In summary, the three factors (counterface type, shear frequency and compressive strain) at each of their two different levels (ball vs. cylinder, 0.2 vs. 1 Hz, 5% vs. 20%) resulted in a 2^3^ (levels^factors^) full factorial design with a center point at 0.6 Hz shear frequency and 10% compressive strain. To differentiate between the loading protocols, we established a naming convention for the loading protocols that can be found in Table 1. Briefly, the number following the "s-" in the name of the loading protocol indicates the shear frequency setting. The number following the "c-" indicates the compressive strain setting.

**Table 1:** Naming convention for the different loading protocols.

Loading protocol	Shear frequency (Hz)	Compressive strain (%)
s-0_2-c-5	0.2	5
s-0_2-c-20	0.2	20
s-0_6-c-10	0.6	10
s-1-c-5	1	5
s-1-c-20	1	20

*s* shear frequency, *c* compressive strain.

Supplementary Fig. 1 shows graphs of the different loading curves that were used in this study. Control scaffolds were kept in unloaded conditions.

### Sample and conditioned medium collection

Conditioned medium was collected and replaced every second day and then stored at − 20 °C for later biochemical analysis. In total, for each scaffold, there were 6 samples of collected conditioned medium (day 0 sample included). Scaffold samples were digested in 1 mL 0.5 mg/mL proteinase K (pK, Roche) at 56 °C for 16 h. The pK reaction was inactivated at 96 °C for 10 min and the samples were stored at − 20 °C.

### Biochemical analyses

#### DNA quantification

The Hoechst 33258 dye (Sigma-Aldrich) was used to quantify the DNA content in pK digested scaffolds. Briefly, 40 µL of each sample, each standard (calf thymus DNA, Sigma-Aldrich) and blank was pipetted into a white-bottom 96-well plate. 160 µL of 1 µg/mL Hoechst dye solution was added and the plate incubated in the dark for 20 min. Fluorescence was measured with excitation at 360 nm and emission at 465 nm using the Victor 3 Micro Plate Reader (Perkin Elmer).

#### Active and latent TGF-β1 and BMP2 quantification

Latent and active TGF-β1 were measured in the conditioned medium using enzyme-linked immunosorbent assay (ELISA) with the TGF-β1 DuoSet ELISA kit (R&D Systems). The active TGF-β1 was detected by adding conditioned medium to the plates. To measure the total produced TGF-β1, the conditioned medium was first acidified and then neutralized to activate the latent TGF-β1, before adding the sample to the plate. Therefore, the total produced TGF-β1 consisted of values from the latent and already active TGF-β1 within the conditioned medium. BMP2 content was measured using an ELISA with the BMP2 DuoSet ELISA kit (R&D Systems), according to manufacturer's instructions. For both ELISAs, a Victor 3 Micro Plate Reader was used to measure the absorbance at 450 nm and 560 nm. The reading at 560 nm was subtracted from the reading at 450 nm and the concentration calculated by fitting a four-parameter logistic curve.

#### sGAG quantification

The 1,9-dimethylmethylene blue (DMMB) assay was used to quantify the sGAG content in the conditioned medium and pK digested samples. Chondroitin 4-sulfate sodium salt from bovine trachea (Fluka) was used as standard, with the highest standard concentration being 1.25 μg/well. Absorbance at 530 nm was measured using the Victor 3 Micro Plate Reader. The DMMB solution was prepared according to Farndale et al.^27^.

#### Nitrite quantification

Nitrite was used as an indirect marker of NO and measured using the Griess Assay (Promega). The assay was performed according to manufacturer's instructions and the absorbance was measured at 530 nm.

### Statistical analysis and design of experiment (DOE)

Statistical analysis was performed using R (version 4.0.2 (2020-06-22))^28^ within the integrated development environment (IDE) RStudio (version 1.3.959)^29^. The R packages that were used can be found in the “Supplementary information”.

A linear mixed model with donor as random variable was used to investigate main and interaction effects of the two factors: (1) counterface type; and (2) loading protocol (different combinations of shear frequency and compressive strain—summarized in Table 1) on different biological markers. Planned orthogonal contrast analysis was used to test hypothesized differences between pre-selected groups. The contrast for the counterface type, contrast1_counterface_, compared the ball to the cylinder (Table 2). In this set of comparisons, the variable counterface type is coded in a way that one can test the difference between the two counterface types. Within the statistical software R, the ball variable is assigned the weight 0, and the cylinder the weight 1 (it would also be possible to code the variables as − 1 and 1, respectively).

**Table 2:** Contrast and the respective weights for the counterface type.

Counterface type	Contrast1_counterface_
Ball	0
Cylinder	1

For the statistical analysis, the factors shear frequency and compressive strain were combined to the factor loading protocol. Previous experiments by Li et al. indicated that mechano-induced chondrogenesis depends on the amplitude of shear frequency and compressive strain^30^. The previously established knowledge allowed us to set contrasts for the statistical analysis. For the loading protocols, contrasts were split once, either first according to shear (Table 3) or first according to compression (Table 4). Within each contrast (e.g. contrast1_shear_), each loading protocol was assigned a weight. The weight was decided upon according to a predefined hypothesis (e.g. the comparison between low shear (0.2 and 0.6 Hz) and high shear (10 Hz)). Meaning that the loading protocols with low shear are grouped and coded the same first value and the loading protocols with high shear are grouped and coded the same second value. For each contrast, the sum of its weights must equal zero. This procedure is repeated until every group only consists of two different loading protocols. Furthermore, if the product of the weights of every condition (in this case, the different loading protocols) adds to zero, the contrasts is orthogonal or independent^31^. This has the advantage that the resulting p-values and regression coefficients are uncorrelated.

**Table 3:** Contrasts and their respective weights for the loading protocols—split first according to shear.

Loading protocol	Contrast1_shear_	Contrast2_shear_	contrast3_shear_	Contrast4_shear_
s-0_2-c-5	− 1	− 1	− 1	0
s-0_2-c-20	− 1	2	0	0
s-0_6-c-10	− 1	− 1	1	0
s-1-c-5	1.5	0	0	− 1
s-1-c-20	1.5	0	0	1

*s* shear frequency, *c* compressive strain.

**Table 4:** ﻿Contrasts and their respective weights for the loading protocols—split first according to compression.

Loading protocol	Contrast1_compr_	Contrast2_compr_	Contrast3_compr_	Contrast4_compr_
s-0_2-c-5	− 1	− 1	− 1	0
s-0_2-c-20	1.5	0	0	− 1
s-0_6-c-10	− 1	− 1	1	0
s-1-c-5	− 1	2	0	0
s-1-c-20	1.5	0	0	1

*s* shear frequency, *c* compressive strain.

For the contrasts that were first split according to shear, this resulted in the first contrast, contrast1_shear_ comparing low shear (0.2 and 0.6 Hz) to high shear (10 Hz). The second contrast, contrast2_shear_ compared low compression (5% and 10%) to high compression (20%) in the low shear (0.2 and 0.6 Hz) group. Contrast3_shear_ compared 5–10% compression in the low shear group. Contrast4_shear_ compared 5–20% compression in the high shear (1 Hz) group.

Alternatively, for the contrasts that were first split according to compression, this resulted in the first contrast, contrast1_compr_ comparing low compression (5% and 10%) to high compression (20%). The second contrast, contrast2_compr_ compared low shear (0.2 and 0.6 Hz) to high shear (1 Hz) in the low compression (5% and 10%) group. Contrast3_compr_ compared 0.2–0.6 Hz shear in the low compression group (5% and 10%). Contrast4_compr_ compared 0.2–1 Hz shear in the high compression (20%) group. All measured values were normalized to the DNA content of the respective scaffolds. The samples were run in duplicates (e.g. there were two scaffold samples for the following characteristics: donor: 1; type of counterface: cylinder; loading protocol: s-0_2-c-5). Comparisons between treatments were made between the sums of the values of each day, resulting in a cumulative end-point value.

The Wilcoxon rank sum test was used to test the difference between load type (loaded vs. unloaded) and the values represent mean ± standard error of the mean (SEM). Effect size was calculated according to Rosenthal^32^.

## Results

### Effect of loading parameters on DNA content

**Figure 2:**
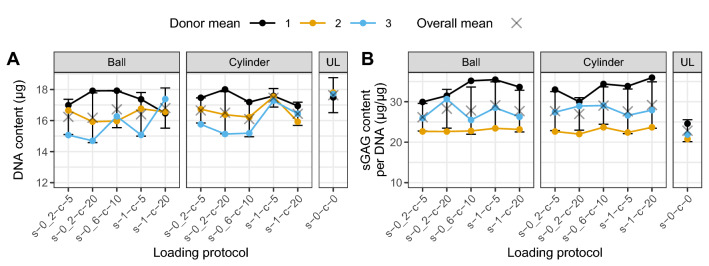
DNA and total sGAG produced (released and retained) content of MSCs seeded in fibrin polyurethane scaffolds and after 10 load cycles.DNA content (**A**) and total sGAG content (sample + medium) (**B**) of MSCs in loaded scaffolds distinguished by counterface type (Ball and Cylinder) or unloaded (UL) condition and loading protocols. Data are shown as mean ± SEM of three independent experiments with cells from three individual donors. Each experimental group was run in technical replicates. Statistical testing was not performed on single conditions but on contrasts (Supp. Fig. 2). *s* shear, *c* compression.

There was a statistically significant difference (*p* < .05, *r* = -.26) in DNA content in loaded scaffolds (6.2% lower, mean = 16.53 μg, SEM = 0.14) after 12 days compared to unloaded controls (mean = 17.63 μg, SEM = 0.38—Supp. Fig. 2A). Contrast analysis revealed that neither counterface type, nor the loading protocols, nor their interaction (Supp. Fig. 3A–H) significantly affected DNA content. Figure 2A shows the DNA content separated by counterface type and loading protocol.

### sGAG response remains unaffected by the choice of loading protocol and counterface

Significantly more total (retained and released) sGAG was produced by loaded scaffolds in comparison to unloaded controls (loaded: 26.02 μg/μg ± 0.55; unloaded: 20.78 μg/μg ± 0.81, *p* < .01, *r* = − .35—Supp. Fig. 2B). However, no significant difference between the loading protocols, the different counterface types, or their interaction was found (Supp. Fig. 3I–P). Furthermore, no statistical difference was observed between unloaded and loaded scaffolds, when measuring only the sGAG retained in the scaffold (data not shown). Figure 2B shows the sGAG content separated by counterface type and loading protocol.

### TGF-β1 is mediated by interactive effects of loading parameters

**Figure 3:**
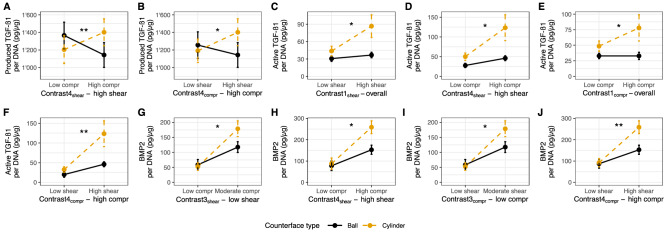
Interaction plots of significant contrasts split first by shear (**A**, **C**, **D**, **G**, **H**) and by compression (**B**, **E**, **F**, **I**, **J**) for produced and active TGF-β1 and BMP2 content. Data are shown as mean ± SEM of three independent experiments with cells from three individual donors. Each experimental group was run in technical replicates. Different loading conditions summarized in contrast groups according to Tables 3 and 4. Significance of interaction between counterface type and contrast groups tested using a linear mixed model with donor as random effect: *p < .05.

**Figure 4:**
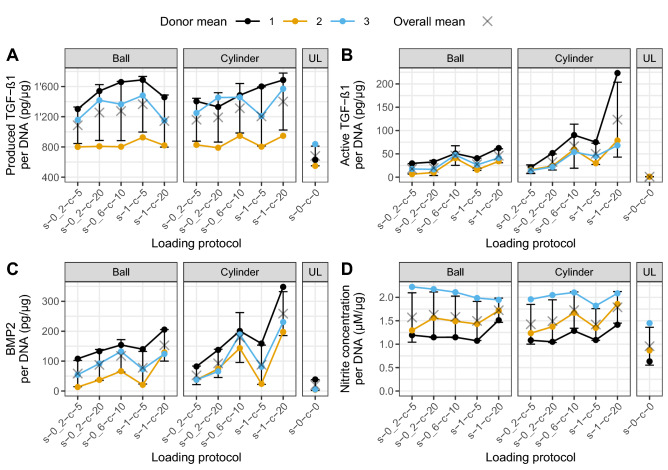
TGF-β1 produced (latent and active), active TGF-β1, BMP2 and nitrite content released by MSCs seeded in fibrin polyurethane scaffolds after 10 load cycles. Produced (**A**) or active (**B**) TGF-β1, BMP2 (**C**) and nitrite (**D**) released by MSCs in loaded scaffolds distinguished by counterface type (Ball and Cylinder) or unloaded (UL) condition and loading protocols. Data are shown as mean ± SEM of three independent experiments with cells from three individual donors. Each experimental group was run in technical replicates. Statistical testing was not performed on single conditions but on contrasts (significant contrasts in Fig. 3; non-significant contrasts in Supp. Figs. 4 and 5). *s* shear, *c* compression.

The total produced TGF-β1 content, consisting of both latent and active TGF-β1, was almost doubled (× 1.83) by loading the scaffolds (loaded: 1245.62 pg/μg ± 40.98; unloaded: 680.77 pg/μg ± 43.44, *p* < .0001, *r* = − .49—Supp. Fig. 2C). There were no significant main effects for either counterface type or loading protocol. However, there was a significant interaction effect of the type of counterface and the loading protocol (*p* < .05). When choosing the cylinder and a loading protocol with high shear frequency (1 Hz), varying from low (5% and 10%) to high (20%) compressive strain significantly increased TGF-β1 production (*p* < .01, contrast4_shear_—Fig. 3A). Similarly, choosing the cylindrical counterface at high compressive strain (20%) and increasing the shear frequency from low (0.2 Hz and 0.6 Hz) to high (1 Hz) significantly increased TGF-β1 production (*p* < .05, contrast4_compr_—Fig. 3B). Therefore, for TGF-β1 production, the cylinder and high shear and high compression are the most favorable factor combination. Figure 4A shows the produced TGF-β1 content separated by counterface type and loading protocol.

Loading the scaffolds activated latent TGF-β1 (46.07 pg/μg ± 5.24). Except for one sample, where a very low amount of active TGF-β1 was detected (which was 115 times lower than the average of the loaded group), no activation was measured in the unloaded controls (Supp. Fig. 2D). For active TGF-β1, there were significant main effects for both counterface type (*p* < .01) and loading protocol (*p* < .001). Most importantly, there was a significant interaction effect of the type of counterface and the loading protocol (*p* < .05). This indicates that the effect of different loading protocols was significantly affected by counterface type. Our hypothesis that the cylinder counterface, with its larger surface, would increase TGF-β1 activation through increased shear compared to the ball counterface was confirmed (*p* < .05, contrast1_shear_—Fig. 3C). Furthermore, planned orthogonal contrast analysis revealed that when using the cylindrical counterface at high shear (1 Hz) the choice of compression significantly affected TGF-β1 activation. In fact, by increasing the compression from 5 to 20% strain, a higher active TGF-β1 content was measured in the medium with a cylinder compared to a ball (*p* < .05, contrast4_shear_—Fig. 3D). By using a cylinder and splitting the contrasts first amongst low (5% and 10%) and high compression (20%), it was shown that loading protocols with high compression increased active TGF-β1 (*p* < .05, contrast1_compr_—Fig. 3E). If high shear (1 Hz) was used with high compression (20%), more activated TGF-β1 was measured (*p* < .01, contrast4_compr_—Fig. 3F). Choosing the cylindrical counterface and the loading protocol with highest shear and compression settings leads to the highest amount of active TGF-β1. Figure 4B shows the active TGF-β1 content separated by counterface type and loading protocol.

### BMP2 content reflects active TGF-β1 content

Loading the scaffolds led to an almost twofold increase in BMP2 compared to unloaded control (loaded: 1245.62 pg/μg ± 40.98; unloaded: 680.77 pg/μg ± 43.44, *p* < .0001, *r* = -.46—Supp. Fig. 2E). Both main effects of counterface type (*p* < .05) and loading protocol (*p* < .001) and most importantly, their interaction (*p* < .01) were significant. Contrast analysis revealed that using a cylinder and applying low shear significantly increased BMP2 content when varying the compression from 5 to 10% strain (*p* < .05, contrast3_shear_—Fig. 3G). Likewise, using a cylinder, applying high shear and changing from 5 to 20% compression led to significant elevation in BMP2 levels (*p* < .05, contrast4_shear_—Fig. 3H). For low compression (5% and 10% strain), already increasing the shear from 0.2 to 0.6 Hz led to an increase in BMP2 when using a cylinder (*p* < .05, contrast3_compr_—Fig. 3I). Also, for high compression (20%), and increase in shear from 0.2 to 1 Hz and selecting the cylindrical counterface resulted in the highest BMP2 content (*p* < .01, contrast4_compr_—Fig. 3J). Figure 4C shows the BMP2 content separated by counterface type and loading protocol.

### Compression affects nitrite response

Similarly, higher nitrite concentration was detected in this study when comparing loaded to unloaded scaffolds (loaded: 1.59 pg/μg ± 0.05; unloaded: 0.96 pg/μg ± 0.14, *p* < .0001, *r* = − .40—Supp. Fig. 2F). While the counterface type did not affect nitrite concentration, contrast analysis revealed that the main effect of loading protocols was significant (*p* < .001). Contrast analysis revealed that at high shear, increasing compression from 5 and 10% to 20% resulted in an increase in nitrite levels (*p* < .05). Additionally, irrespective of shear frequency, increasing compression from low (5% and 10%) to high (20%) lead to a significant increase in nitrite (*p* < .05). The interaction between counterface type and loading protocol did not significantly alter measured nitrite concentration (Supp. Fig. 5). Figure 4D shows the nitrite concentration separated by counterface type and loading protocol.

### Correlation of the different response variables

**Figure 5:**
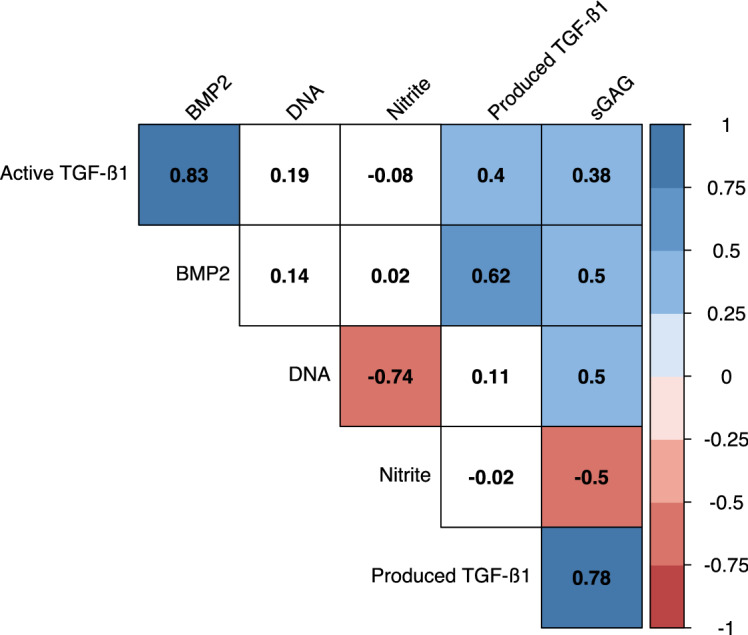
Correlogram of the different response variables. Data from three independent experiments with cells from three individual donors. Each experimental group was run in technical replicates. All response variables are normalized to the DNA content (except the DNA content). Number in squares refers to the Pearson's r correlation coefficient. *p* < 0.05 for all squares that are not blank. Red colors refer to negative correlation, blue colors refer to positive correlation. Supp. Fig. 6 shows the correlograms according to the different loading protocols.

Amongst the loading conditions, the measured BMP2 correlated strongly with the amount of activated TGF-β1 (*r* = .83, *p* < .05—Fig. 5). Similarly, total sGAG content appeared to strongly correlate with total produced TGF-β1 (*r* = .78, *p* < .05—Fig. 5). However, the DNA content within the loaded scaffolds was inversely correlated with the nitrite concentration (*r* = − .74, *p* < .05—Fig. 5). Splitting the correlogram by the different loading protocols did not appear to drastically change the relationships between the different response variables (Supp. Fig. 6).

## Discussion

Clinicians and researchers place significant hope on TE to regenerate tissues with limited potential for self-repair. Progenitor cells such as MSCs, play a key role, given their potential for both proliferation and differentiation. Regarding the latter, an adequate physical environment is believed to be a requirement for successful differentiation^8,33^. However, the exact mechanism through which MSCs differentiate remains unclear. A streamlined way to test multiple conditions could offer new insights into the mechano-induced differentiation of MSCs and accelerate the identification of suitable biomaterials for cartilage and bone repair.

In this study, MSCs seeded within fibrin-PU scaffolds were exposed to joint-mimicking multiaxial compression and shear, a complex mechanical stimulation known to drive chondrogenesis^22,23,26^. By combining a full factorial DOE with contrasts and a linear mixed model, we aimed to test different loading parameters by measuring different candidate biomarkers secreted by the MSCs. This approach demonstrated interactive effects between counterface type and loading protocols on biological marker production and could therefore prove useful for the investigation of possible interactions across certain factors when testing biomaterials.

We previously showed that mechanical stimulation in our bioreactor system activates latent TGF-β1^11^. Multiple reports have highlighted that fluid shearing plays a role in the production and activation of latent TGF-β^34–36^. Additionally, integrin binding to latent TGF-β1 that is linked to the ECM has been demonstrated to result in active TGF-β1 release^37^. We surmise that fluid shearing mainly activates the latent TGF-β1 during the early stages, where no ECM is present. This is in line with research from Tanaka et al. that suggests fluid shearing to be the dominant effect during compression of porous scaffolds^38^. On the other hand, at later stages, when mature ECM is present, the latent ECM-bound TGF-β1 is released and activated by the local deformation of the scaffold. In our study, the increase in both shear and compression coupled with an increase in scaffold surface exposed to maximal compression combined with shear due to the counterface type (cylinder) promoted TGF-β1 activation, a main driver of chondrogenesis. These findings have previously been confirmed by gene expression results investigating chondrogenesis in MSCs^30^. Additionally, Zahedmanesh et al*.* used a finite element model to show that local compressive deformations, with negligible pore fluid pressure suffice to induce chondrogenesis in the same MSC enriched fibrin-PU scaffolds^39^. They also showed that the combination of compression and interfacial shear lead to the highest minimum magnitude in all three compressive principal strains as opposed to shear or compression alone^39^. We have previously shown that mechanical activation of TGF-β can occur in the absence of cells^11^. Therefore, we reason that the cylindrical counterface surface, together with the increased magnitude of compression and shear, enhanced the local activation of TGF-β. As the pericellular matrix develops and matures, the larger compressive deformation may further increase TGF-β1 activation via integrin-mediated tension on the cells.

BMP2, which is another member of the TGF-β superfamily, showed very similar trends to the TGF-β1 measured in the medium. In fact, BMP2 has been shown to respond to tensile stress in a rat osteotomy study^40^. Another study showed enhancing effects of mechanical signals in the BMP signaling pathway^41^. However, it is debatable whether increased BMP2 in our experiment would eventually improve chondrogenesis. Not only is BMP2 an important marker for chondrogenesis, but also for osteogenesis. BMP2 plays a role in fracture repair and in the induction of chondrocyte hypertrophy^42,43^. Additionally, BMP2 has been shown to autoregulate its expression in mice^44^. This suggests that BMP2 could also be increased via a positive feedback mechanism.

The combination of BMP2 and TGF-β1 has been demonstrated to be more effective in inducing chondrogenesis than TGF-β1 alone in rabbit bone marrow derived MSCs^45^. Additionally, it was observed that gene expression of the hypertrophic marker collagen type X was not significantly increased by using a loading protocol combining compression and shear^26^. However, the exact mechanism how TGF-β1 and BMP2 interact is currently unknown. Elevated levels of active TGF-β1 may potentially lead to increased BMP2 content. Their dependency could be explored by blocking the TGF-β1 with TGF-β receptor 1 (ALK5) inhibitor LY364947, as already successfully performed by Li et al. in our system^46^. Our DOE approach would then be a useful tool to correlate different concentrations of TGF-β1 and BMP2 to the histological outcomes.

In light of the increased amount of active TGF-β1 in specific loading protocols, one would also assume the MSCs to be pushed towards chondrogenesis, resulting in an increase in sGAG production in loaded samples. Indeed, significantly more sGAGs were measured in the medium of the loaded samples compared to unloaded controls, however the sGAGs measured in the samples did not significantly differ. This suggests that while total GAG production increased, the short culture time of 12 days was not sufficient for the scaffolds to produce enough matrix to retain the elevated sGAG content that was induced by the activated TGF-β1 in the loaded groups.

The emphasis of this experiment was to use a DOE design to reduce the number of candidate loading conditions for subsequent longer experiments. Previous studies performed by our group have shown that early data accurately represents longer-term results, thus enabling an early rapid screening approach^11^. Furthermore, multiaxial load favors a chondrogenic phenotype^30^ and the expression of TGF-β1 in the medium correlates with sGAG production by the cells, further validating its use as a predictive marker^47^. Nevertheless, chondrogenesis experiments using the identified conditions will be performed for longer periods of at least 21 days as it usually takes several weeks until enough mature matrix has formed that could retain observable amounts of chondrogenic proteins. Longer studies with fewer groups selected from this successful screen, with histology and immunohistochemistry as end-point measurements are planned to validate our short-term findings.

Nitric oxide and its indirect marker nitrite have been suggested to respond to mechanical load via inducible nitric oxide synthase (iNOS)^48^. Similarly, in our study, higher nitrite concentration was measured in loaded scaffolds compared to controls. However, even though higher mechanical load could potentially lead to larger stress on the cells, increasing the amplitude of loading protocols did not statistically increase the nitrite production. Correlation analysis highlighted that increase nitrite negatively correlated with DNA and sGAG content. Increased amplitude did not further increase nitrite production but did increase TGF-β1activation. In particular, the cylindrical counterface and the loading protocol with highest shear and compression settings led to the highest amount of active TGF-β1. This indicates the loading conditions can be optimized to increase TGF-β1activation, without further increasing the negative nitrite effects.

In unloaded controls, the cells seeded on top of the scaffolds remain undisturbed from mechanical load, which might be more permissive to proliferation. Therefore, we hypothesize that these undisturbed top-layer cells accounted for the higher DNA content in the unloaded scaffolds.

In the TE field, differences amongst treatment methods are usually investigated using cells from different donors. The heterogeneity amongst donors already accounts for a very large part of variability, therefore the effect of different treatment methods, which represents the introduced systematic variability, is often concealed^49^. This could mean that despite the existence of similar trends amongst all donors, actual differences are obfuscated by averaging the values among the donors^26^. It seems sensible to expect output values stemming from different treatment levels of the same donor to be more similar to each other than they are to values stemming from treatment levels of another donor. This would identify the general trends, which would then establish the general effect of a treatment, irrespective of donor. Furthermore, in tissue engineering, the focus has shifted towards patient specific treatments and in the clinical setting, stem cells from different donors are also not pooled. Using linear mixed models that block for donors could better describe the reality than simple ANOVAs and more work should be done in this direction to validate this hypothesis.

Additionally, the use of planned contrasts allows for predetermined comparisons between conditions or clusters of conditions to test specific hypotheses. In experiments that compare more than two groups, methods such as ANOVAs cannot explain which groups are different from each other. While, subsequent t-tests are par for the course, they lose statistical power by not considering the complete data and need to be corrected for multiple comparisons^50^. Planned contrasts allow for a priori defined comparisons to be directly tested.

With the DOE, we were able to show the application of a methodological framework that allows the delineation of complex interactions between factors. This can be utilized to assess optimal mechanical loading protocols with the longer-term aim of evidence-based rehabilitation protocols, further building on the concept of regenerative rehabilitation. Furthermore, in a field like TE, where new biomaterials with manifold properties are continuously being developed, a fast screening using this method could provide insights on whether a biomaterial will prove suitable in the future.

### Supplementary Information

Supplementary Figures.

## Data Availability

Relevant data is contained within the manuscript. Additional data can be provided upon reasonable request.

## Appendix

R packages used:

- tidyverse
- magrittr
- lme4
- lmerTest
- corrplot
- pastecs
- devEMF
- Scales
- Base packages

## Abbreviations

αMEM: Minimum essential medium alpha
ANOVA: Analysis of variance
BMP2: Bone morphogenetic protein 2
CpM: Chondropermissive medium
DMEM: Dulbecco’s modified Eagle medium
DMMB: 1,9-Dimethylmethylene blue
DNA: Deoxyribonucleic acid
DOE: Design of experiment
ELISA: Enzyme linked immunosorbent assay
FGF-b: Fibroblast growth factor basic protein
sGAG(s): Sulphated glycosaminoglycan(s)
IDE: Integrated development environment
iNOS: Inducible nitric oxide synthase
MSCs: Mesenchymal stem/stromal cells
NO: Nitric oxide
OFAT: One Factor at a Time
pK: Proteinase K
PU: Poly(ester-urethane)
SEM: Standard error of the mean
TE: Tissue engineering
TGF-β1: Transforming growth factor β1

## References

[1] Haas NP (2000). Callus modulation—Fiction or reality?. Chirurg.

[2] Delaine-Smith RM, Reilly GC (2012). Mesenchymal stem cell responses to mechanical st`imuli. Muscles Ligaments Tendons J..

[3] Fahy N, Alini M, Stoddart MJ (2018). Mechanical stimulation of mesenchymal stem cells: Implications for cartilage tissue engineering. J. Orthop. Res..

[4] Luo ZJ, Seedhom BB (2007). Light and low-frequency pulsatile hydrostatic pressure enhances extracellular matrix formation by bone marrow mesenchymal cells: An in-vitro study with special reference to cartilage repair. Proc. Inst. Mech. Eng. H.

[5] Steadman JR, Rodkey WG, Rodrigo JJ (2001). Microfracture: Surgical technique and rehabilitation to treat chondral defects. Clin. Orthop. Relat. Res..

[6] Buckwalter JA, Mankin HJ (1998). Articular cartilage repair and transplantation. Arthritis Rheum..

[7] Hunziker EB (2002). Articular cartilage repair: Basic science and clinical progress. A review of the current status and prospects. Osteoarthritis Cartil..

[8] Erggelet C, Vavken P (2016). Microfracture for the treatment of cartilage defects in the knee joint—A golden standard?. J. Clin. Orthop. Trauma.

[9] Johnstone B, Hering TM, Caplan AI, Goldberg VM, Yoo JU (1998). In vitro chondrogenesis of bone marrow-derived mesenchymal progenitor cells. Exp. Cell Res..

[10] Barry F, Boynton RE, Liu B, Murphy JM (2001). Chondrogenic differentiation of mesenchymal stem cells from bone marrow: Differentiation-dependent gene expression of matrix components. Exp. Cell Res..

[11] Li Z, Kupcsik L, Yao SJ, Alini M, Stoddart MJ (2010). Mechanical load modulates chondrogenesis of human mesenchymal stem cells through the TGF-beta pathway. J. Cell Mol. Med..

[12] Behrendt P (2020). Articular joint-simulating mechanical load activates endogenous TGF-beta in a highly cellularized bioadhesive hydrogel for cartilage repair. Am. J. Sports Med..

[13] Sumanasinghe RD, Bernacki SH, Loboa EG (2006). Osteogenic differentiation of human mesenchymal stem cells in collagen matrices: Effect of uniaxial cyclic tensile strain on bone morphogenetic protein (BMP-2) mRNA expression. Tissue Eng..

[14] Jaiswal N, Haynesworth SE, Caplan AI, Bruder SP (1997). Osteogenic differentiation of purified, culture-expanded human mesenchymal stem cells in vitro. J. Cell Biochem..

[15] Halvorsen YD (2001). Extracellular matrix mineralization and osteoblast gene expression by human adipose tissue-derived stromal cells. Tissue Eng..

[16] Hofseth LJ (2003). Nitric oxide-induced cellular stress and p53 activation in chronic inflammation. Proc. Natl. Acad. Sci. USA.

[17] Forstermann U, Xia N, Li H (2017). Roles of vascular oxidative stress and nitric oxide in the pathogenesis of atherosclerosis. Circ. Res..

[18] Fox SW, Chambers TJ, Chow JW (1996). Nitric oxide is an early mediator of the increase in bone formation by mechanical stimulation. Am. J. Physiol..

[19] Montgomery DC (2013). Design and Analysis of Experiments.

[20] Oehlert, G. W. *A First Course in Design and Analysis of Experiments*. 2010. http://users.stat.umn.edu/~gary/book/fcdae.pdf. Accessed 10 August 2020.

[21] Gardner OF, Alini M, Stoddart MJ (2015). Mesenchymal stem cells derived from human bone marrow. Methods Mol. Biol. (Clifton, N.J.).

[22] Li Z, Kupcsik L, Yao SJ, Alini M, Stoddart MJ (2009). Chondrogenesis of human bone marrow mesenchymal stem cells in fibrin-polyurethane composites. Tissue Eng. Part A.

[23] Gardner OFW (2017). Asymmetrical seeding of MSCs into fibrin-poly(ester-urethane) scaffolds and its effect on mechanically induced chondrogenesis. J. Tissue Eng. Regen. Med..

[24] Wimmer MA (2004). Tribology approach to the engineering and study of articular cartilage. Tissue Eng..

[25] Gardner OFW, Fahy N, Alini M, Stoddart MJ (2017). Joint mimicking mechanical load activates TGFbeta1 in fibrin-poly(ester-urethane) scaffolds seeded with mesenchymal stem cells. J. Tissue Eng. Regen. Med..

[26] Schatti O (2011). A combination of shear and dynamic compression leads to mechanically induced chondrogenesis of human mesenchymal stem cells. Eur. Cell Mater..

[27] Farndale RW, Buttle DJ, Barrett AJ (1986). Improved quantitation and discrimination of sulphated glycosaminoglycans by use of dimethylmethylene blue. Biochim. Biophys. Acta.

[28] R: A Language and Environment for Statistical Computing (2020).

[29] RStudio: Integrated Development for R. RStudio (RStudio, PBC, 2020).

[30] Li Z, Yao SJ, Alini M, Stoddart MJ (2010). Chondrogenesis of human bone marrow mesenchymal stem cells in fibrin-polyurethane composites is modulated by frequency and amplitude of dynamic compression and shear stress. Tissue Eng. Part A.

[31] Field AP, Miles J, Field Z (2012). Discovering Statistics Using R.

[32] Rosenthal R (1991). Meta-Analytic Procedures for Social Research.

[33] Steward AJ, Kelly DJ (2015). Mechanical regulation of mesenchymal stem cell differentiation. J. Anat..

[34] Ahamed J (2008). In vitro and in vivo evidence for shear-induced activation of latent transforming growth factor-beta1. Blood.

[35] Albro MB (2012). Shearing of synovial fluid activates latent TGF-beta. Osteoarthritis Cartil..

[36] Ohno M, Cooke JP, Dzau VJ, Gibbons GH (1995). Fluid shear stress induces endothelial transforming growth factor beta-1 transcription and production. Modulation by potassium channel blockade. J. Clin. Invest..

[37] Wipff PJ, Rifkin DB, Meister JJ, Hinz B (2007). Myofibroblast contraction activates latent TGF-beta1 from the extracellular matrix. J. Cell Biol..

[38] Tanaka SM (2005). Osteoblast responses one hour after load-induced fluid flow in a three-dimensional porous matrix. Calcif. Tissue Int..

[39] Zahedmanesh H (2014). Deciphering mechanical regulation of chondrogenesis in fibrin-polyurethane composite scaffolds enriched with human mesenchymal stem cells: A dual computational and experimental approach. Tissue Eng. Part A.

[40] Sato M (1999). Mechanical tension-stress induces expression of bone morphogenetic protein (BMP)-2 and BMP-4, but not BMP-6, BMP-7, and GDF-5 mRNA, during distraction osteogenesis. J. Bone Miner. Res..

[41] Kopf J, Petersen A, Duda GN, Knaus P (2012). BMP2 and mechanical loading cooperatively regulate immediate early signalling events in the BMP pathway. BMC Biol..

[42] D'Angelo M (2000). MMP-13 is induced during chondrocyte hypertrophy. J. Cell Biochem..

[43] Tsuji K (2006). BMP2 activity, although dispensable for bone formation, is required for the initiation of fracture healing. Nat. Genet..

[44] Ghosh-Choudhury N (2001). Autoregulation of mouse BMP-2 gene transcription is directed by the proximal promoter element. Biochem. Biophys. Res. Commun..

[45] Nasrabadi D, Rezaeiani S, Eslaminejad MB, Shabani A (2018). Improved protocol for chondrogenic differentiation of bone marrow derived mesenchymal stem cells-effect of PTHrP and FGF-2 on TGFbeta1/BMP2-induced chondrocytes hypertrophy. Stem Cell Rev. Rep..

[46] Li Z, Kupcsik L, Alini M, Yao SJ, Stoddart M (2010). Mechanical load modulates chondrogenesis of human mesenchymal stem cells through the TGF-beta pathway. J. Cell Mol. Med..

[47] Kupcsik L, Stoddart MJ, Li Z, Benneker LM, Alini M (2010). Improving chondrogenesis: Potential and limitations of SOX9 gene transfer and mechanical stimulation for cartilage tissue engineering. Tissue Eng. Part A.

[48] Gardner OF, Fahy N, Alini M, Stoddart MJ (2016). Differences in human mesenchymal stem cell secretomes during chondrogenic induction. Eur. Cell Mater..

[49] Stoddart MJ, Richards RG, Alini M (2012). In vitro experiments with primary mammalian cells: To pool or not to pool?. Eur. Cell Mater..

[50] Schad DJ, Vasishth S, Hohenstein S, Kliegl R (2020). How to capitalize on a priori contrasts in linear (mixed) models: A tutorial. J. Memory Lang..

