# Supplementary material for: Optimization of loading protocols for tissue engineering experiments
Source: Sci Rep. 2022 Mar 24;12:5094. doi: 10.1038/s41598-022-08849-y (PMC8948220; doi:10.1038/s41598-022-08849-y)
Supplement: Supplementary file 1 — Supplementary Figures. [file 41598_2022_8849_MOESM1_ESM.docx]

# Supplementary figures

Supplementary Figure 1

Graphs of the selected loading protocols. For each graph, the top row depicts excerpts of the oscillatory rotation (±25°) of the counterface at a frequency (Hz). The bottom row depicts the cyclic vertical translational movement of the counterface inducing a compressive strain (%). The loading protocols are in the following order (refer to Table 1): (A) s-0_2-c-5 (*e.g.* 0.2 Hz and 5%), (B) s-0_2-c-20, (C) s-1-c-5, (D) s-1-c-20, (E) s-0_6-c-10.

Supplementary Figure 2

Different biological marker contents of MSCs in unloaded and loaded (average of different loading protocols with each counterface) scaffolds. A: DNA, B: total sGAG (released and retained in scaffold), C: produced TGF-β1, D: active TGF-β1, E: BMP2, F: nitrite. Data are shown as mean±SEM of three independent experiments with cells from three individual donors. Each experimental group was run in technical replicates. Difference tested using the Wilcoxon rank sum test. **p* < .05, ***p* < .01, ****p* < .001, *****p* < .0001.

Supplementary Figure 3

Interaction plots of the contrasts split first by shear (A-D) and by compression (E-H) for the DNA content and interaction plots of the contrasts split first by shear (I-L) and by compression (M-P) for the total sGAG (sample + medium) content. Data are shown as mean±SEM of three independent experiments with cells from three individual donors. Each experimental group was run in technical replicates. Different loading conditions summarized in contrast groups according to Table 3 and Table 4. Significance of interaction between counterface type and contrast groups tested using a linear mixed model with donor as random effect: **p* < .05.

Supplementary Figure 4

Interaction plots of non-significant contrasts split first by shear (A, B, C, G, H, K, L) and by compression (D, E, F, I, J, M, N) for produced and active TGF-β1 and BMP2 content**.** Data are shown as mean±SEM of three independent experiments with cells from three individual donors. Each experimental group was run in technical replicates. Different loading conditions summarized in contrast groups according to Table 3 and Table 4. Significance of interaction between counterface type and contrast groups tested using a linear mixed model with donor as random effect: **p* < .05.

Supplementary Figure 5

Interaction plot of the contrasts split first by shear (A-D) and by compression (E-H) for the nitrite content. Data are shown as mean±SEM of three independent experiments with cells from three individual donors. Each experimental group was run in technical replicates. Different loading conditions summarized in contrast groups according to Table 3 and Table 4. Significance of interaction between counterface type and contrast groups tested using a linear mixed model with donor as random effect: **p* < .05.

Supplementary Figure 6

Correlogram of the different response variables split by loading protocol. Data from three independent experiments with cells from three individual donors. Each experimental group was run in technical replicates. All response variables are normalized to the DNA content (except the DNA content). Number in squares refers to the Pearson's r correlation coefficient. *p* < .05 for all squares that are not blank. Red colors refer to negative correlation, blue colors refer to positive correlation.
